# Case report: A case of heterogeneity of the antitumor response to immune checkpoint inhibitors in a patient with relapsed hepatocellular carcinoma

**DOI:** 10.3389/fonc.2022.899811

**Published:** 2022-07-29

**Authors:** Yingying Bao, Liang Wen, Wen Chen, Jianhui Zhao, Yixiao Yang, Tao Wei, Jian Zhang, Tingbo Liang

**Affiliations:** ^1^ Department of Hepatobiliary and Pancreatic Surgery, The First Affiliated Hospital, Zhejiang University School of Medicine, Hangzhou, China; ^2^ Zhejiang Provincial Key Laboratory of Pancreatic Disease, The First Affiliated Hospital, Zhejiang University School of Medicine, Hangzhou, China; ^3^ The Innovation Center for the Study of Pancreatic Diseases of Zhejiang Province, The First Affiliated Hospital, Zhejiang University School of Medicine, Hangzhou, China

**Keywords:** hepatocellular carcinoma, immune checkpoint inhibitor, lung metastases, α-fetoprotein, complex treatment

## Abstract

The existence of tumor heterogeneity is widely recognized; however, heterogeneity of the antitumor response in multiple tumor nodules in the same patient has not been reported. Sintilimab, a monoclonal antiprogrammed cell death receptor-1 (PD-1) antibody, was used to treat patients with unresectable hepatocellular carcinoma (HCC). In the present study, we report a case of therapeutic heterogeneity in relapsed HCC with lung metastases. A 57-year-old female patient was diagnosed with HCC and underwent radical hepatectomy. One and a half years later, imaging scans found multiple metastatic tumors in the lung, which were accompanied by an increased α-fetoprotein (AFP) level. The patient then started to receive sintilimab. In the first 6 months after sintilimab treatment, all the metastatic nodules regressed gradually and ultimately disappeared, except for one nodule, which remained stable in the following 3 months. Finally, the patient underwent pulmonary lobectomy to remove the remaining nodule. Thereafter, follow-up visits showed the AFP level decreased to normal and imaging scans showed no signs of recurrence, confirming that the patient exhibited a clinically complete response. Pathological assessments showed that in the primary tumor site, the tumor comprised moderately differentiated HCC with a few infiltrated cytotoxic T cells and negative PD-L1 expression. While in the metastatic site, the nodule was composed of poorly differentiated HCC with cytotoxic T-cell infiltration with few cells inside the tumor and expressed PD-L1 in some areas of the tumor. There were dynamic alterations of PD-L1 expression and cytotoxic T-cell infiltration in the primary and relapsed HCC lesions after anti-PD-1 treatment. This case presented the heterogeneities of both the tumor microenvironment and the following antitumor response among the metastatic nodules in the same patient and revealed the importance of comprehensive therapy in cancer treatment.

## Introduction

Tumor heterogeneity, including interindividual, intertumor, and intratumor types, has been widely recognized (1). The antitumor responses to chemotherapy, targeted therapy, or immune therapy among patients also vary widely (2, 3). However, no heterologous effects of these therapies have been observed in one patient with multiple tumor sites.

Sintilimab is a monoclonal antibody that binds to programmed cell death receptor-1 (PD-1), thereby blocking the interaction of PD-1 with its ligands (PD-L1 and PL-L2). Sintilimab has been approved in China for patients with relapsed or refractory Hodgkin’s lymphoma (4). The ORIENT-32 study found that compared to sorafenib monotherapy, a combination of sintilimab and IBI305 (a bevacizumab biosimilar) showed significantly prolonged survival as a first-line treatment for unresectable HCC (5). In the present study, we report a case of therapeutic heterogeneity of sintilimab in the treatment of relapsed HCC with lung metastases and report the follow-up treatments.

## Case report

In November 2018, a 57-year-old female patient was admitted to our hospital because of tumor relapse with multiple lung metastases after hepatectomy of HCC over 2 years ago. She had no chronic viral hepatitis, fatty liver, liver cirrhosis, or any liver diseases. Furthermore, she had no history of long-term drinking, diabetes mellitus, autoimmune diseases, or any other underlying disease.

**Figure 1:**
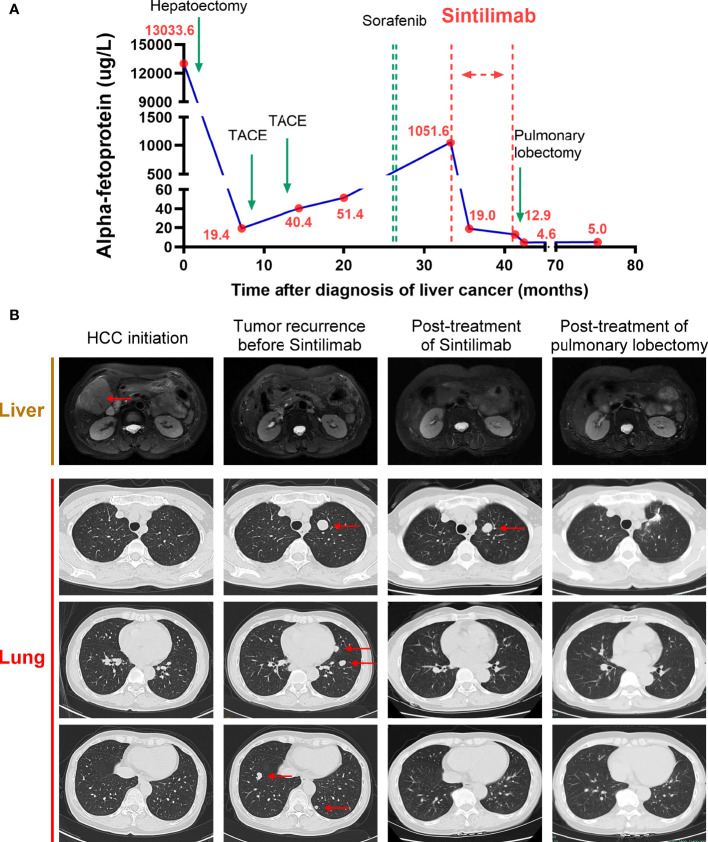
Course of the patient’s treatments. **(A)** Timelines of main treatments and changes in α-fetoprotein (AFP) levels during each period.**(B)** Representative imaging assessments during each period, including hepatic magnetic resonance (T2-weighted) and pulmonary computerized tomography (CT) in three different sections. Red arrows indicate the tumors.

In February 2016, the patient was diagnosed with HCC in liver segments V and VI (6*5 cm) (**Figure 1B**) and underwent hepatectomy with R0 resection. The pathological report showed the surgical margin was tumor negative, and there was no lymph node invasion. Seven months later, the patient received trans-hepatic arterial chemotherapy and embolization (TACE) twice because of suspected tumor recurrence indicated by re-increased α-fetoprotein (AFP) levels (**Figure 1A**). In September 2017, imaging scans revealed multiple relapsed metastatic tumors in the lung but not in the liver (**Figure 1B**). Therefore, the patient was treated with sorafenib in a dose of 400 mg twice daily from April 2018 and was discontinued 3 weeks later due to an intolerable side effect of severe rash. After the cessation of sorafenib treatment, the patient’s AFP level continued to increase (**Figure 1A**).

**Figure 2:**
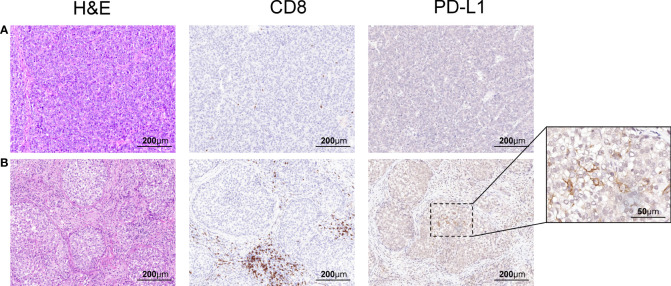
Representative pathological sections of primary and recurrent tumors. **(A)** In the primary tumor in the liver, H&E staining and IHC showed infiltration of a few CD8-positive T cells and negative PD-L1 expression. **(B)** In the remaining tumor in the lung after sintilimab treatment, H&E staining and IHC showed infiltration of CD8-positive T cells in the mesenchymal area but not in the tumor and focal PD-L1 expression.

Beginning in November 2018, she received 200 mg of sintilimab every 3 weeks for 9 months. There was no fatigue, fever, nausea, erythema, or any other severe side effects during treatment. The AFP level soon decreased to 19.0 g/L after three cycles of sintilimab and remained stable between 12.0 to 20 g/L from January 2019 to July 2019 (**Figure 1A**). During follow-up visits, we did not find any tumor recurrence in the abdomen. In the lung, surprisingly, except for one nodule in the superior lobe of the left lung, all the other nodules regressed gradually, and 6 months after sintilimab, they finally disappeared. The single remaining pulmonary nodule neither regressed nor progressed during 9 months of sintilimab treatment (**Figure 1B**). After a full discussion by the multidisciplinary team in our hospital, in July 2019, the patient underwent a pulmonary lobectomy to remove the remaining nodule. The AFP level decreased to normal thereafter. The imaging scans also showed no signs of recurrence in recent months (**Figure 1B**). In subsequent follow-up visits every 3 months, the patient’s AFP remained at normal levels, and no obvious signs of recurrence have been found on imaging. We also performed pathological assessments to further show the heterogeneity between the primary site (**Figure 2A**) and the resected metastatic nodule (**Figure 2B**) after sintilimab treatment. The results showed that in the primary tumor site (**Figure 2A**), the tumor was composed of moderately differentiated HCC with a few infiltrated cytotoxic T cells and negative PD-L1 expression. While in the metastatic site, the nodule (**Figure 2B**) was composed of poorly differentiated HCC with cytotoxic T-cell infiltration, especially in the mesenchymal tissue around the tumor. However, there were few cytotoxic T cells inside the tumor, and PD-L1 expression was observed in some areas of the tumor. Furthermore, regulatory T cells (Tregs) were only identified in the metastatic site while more T and B cells were found in the metastatic site compared to that in the primary tumor site. The infiltration of other immune cells such as macrophages, natural killer cells, and dendritic cells was comparable between these two sites (**Supplementary Figure S1**). All this evidence indicated heterogeneity of tumor development during HCC recurrence and the existence of divergent antitumor effects on different tumor nodules by anti-PD-1 treatment in the same patient.

## Discussion

We reported a case of heterogeneous antitumor efficacy of anti-PD-1 treatment on multiple lung metastatic lesions in a patient with recurrent HCC. Sintilimab treatment resulted in the disappearance of all except one of the metastatic nodules, which was finally removed surgically. By combining immune and surgical therapy, the patient achieved clinically complete remission (CR). Accordingly, we found the different pathological features of tumor differentiation level, cytotoxic T-cell infiltration, and PD-L1 expression in the tumor between the primary site and metastatic site, indicating intertumor heterogeneity along with tumor development.

Currently, the study for tumor heterogeneity demonstrated that there was both spatial heterogeneity and temporal heterogeneity during tumor development (6), and an accurate assessment of tumor heterogeneity usually benefits the judgment of therapeutic effect. A recent study shows that the heterogeneity of recurrent liver cancer contributes to the development of autoimmune paraneoplastic syndrome through antitumor immune responses (7). Some evidence supports a close relationship between primary tumors and metastatic outgrowths (8), while more reports revealed the divergent tumor microenvironment among tumor primary and metastatic sites. In the new era of immunotherapy, PD-L1 expression and cytotoxic T-cell infiltration are regarded as two key factors for assessing tumor heterogeneity. A heterogeneous expression of PD-L1 and infiltration of CD8+ TILs have been reported in several tumors, within the same tumor, and between a primary tumor and its metastasis in the same patient (9, 10). Furthermore, many studies reported there was a positive association between PD-L1 expression in tumor cells and the presence of TILs in melanoma, lung, breast cancer, and other solid tumors (10–12). In this case, we observed dynamic alterations of PD-L1 expression and cytotoxic T cells, i.e., some infiltrated cytotoxic T cells and negative PD-L1 expression in the primary and, on the contrary, few T cells but expressed PD-L1 in the relapsed HCC lesions after sintilimab treatment, which implied heterogeneous microenvironments between the primary tumor and its metastasis in this case. On the other hand, the clonal heterogeneity and immune microenvironments within metastases remain unexplored. One limitation of this study was that it is hard to assess the immune status of other lung metastatic nodules in this case. Due to the existence of divergent efficiency of sintilimab among multiple lung nodules in this patient, it is reasonable to speculate the intermetastases heterogeneity. In a word, this case demonstrated the existence of both spatial heterogeneity and temporal heterogeneity in the same patient.

For the efficacy of anti-PD-1 treatment, increased cytotoxic T-cell infiltration and PD-L1 expression in the tumor microenvironment are always considered two important favorable factors. For PD-L1 expression, it is generally believed that high PD-L1 expression is related to increased response rate and clinical benefit in the anti-PD-1/anti-PD-L1 treatment of many solid tumors (13, 14). However, there are also reports showing that in many cases, tumors classified as PD-L1 negative also respond to treatment (15, 16), and there was no significant difference in observed objective response rate between PD-L1 expression-positive and PD-L1 expression-negative subgroups (17), revealing the complexity to use PD-L1 expression as a predictive marker for antitumor efficacy. On the other hand, the density of cytotoxic T-cell infiltration, or tumor-infiltrating lymphocyte (TIL), has been confirmed to be associated with clinical benefits in the immunotherapy of many cancers (18, 19). When considering both TIL status (presence or absence) and PD-L1 expression status (positive or negative), the tumor type with both TIL presence and PD-L1-positive expression is most likely to respond to PD-1/PD-L1 blockade therapy (20). However, in this case, there was PD-L1-positive expression but a lack of cytotoxic T-cell infiltration in the lung-remaining metastatic tumor, which seems to be the type (PD-L1+TIL−) that was prone to resist monotherapy of PD-1/PD-L1 inhibitors (20), giving the rationale that it did not show any response to sintilimab. CD4+CD25+ Tregs, which express a panel of chemokine receptors and surface molecules such as CTLA4, PD-1, and others, are among the most prevalent suppressor cells in TME. Chemokines secreted by hepatoma cells, such as CCL5, CCL22, and CCL28, increase the infiltration of those Tregs, leading to the impaired activity of antigen-presenting cells (APCs) and immune cells in TME (21). Therefore, it can be speculated that CD4+CD25+ Tregs in different tumor microenvironments are a potential mechanism of the heterologous effects of anti-PD-1 treatment. In this case, the infiltration of Tregs was observed in the remaining lesions of the lung, which was possibly associated with the treatment resistance of PD-1 inhibitors. Additionally, other tumor microenvironment-related biomarkers like IFN-γ signaling, tumor mutational burden, and microsatellite instability, which were not assessed in this patient, could also affect the antitumor efficacy of anti-PD-1 treatment (22).

The treatment of advanced liver cancer has taken a leap forward with the advent of immune checkpoint inhibitors. However, numerous trials have demonstrated heterogeneity in tumor responses to these immune-oncology agents. In the recently completed phase III trial of pembrolizumab (KEYNOTE-240), the overall survival and progression-free survival of patients were not improved compared to that in the control group. The trial showed that only 2% of all subjects experienced CR, while 16% and 32% experienced partial remission (PR) and disease progression (PD), respectively (23). Tumor resistance to immunotherapy is potentially linked to oncogenic pathways and mechanisms. Substantial evidence shows that the Wnt/β-catenin signaling pathway is associated with tumor immune suppression (24). Mechanisms by which tumor-intrinsic active β-catenin signaling results in T-cell exclusion and resistance to anti-PD-L1/anti-CTLA-4 monoclonal antibody therapy were discovered in an orthotopic mouse model of melanoma. This study demonstrates that the Wnt/β-catenin signaling pathway induces the expression of the transcriptional repressor ATF3, resulting in decreased CCl4 gene expression and ultimately the defective recruitment of CD103+ dendritic cells in the tumor microenvironment (25). β-Catenin activation promotes immune escape and resistance to anti-PD-1, as validated in a novel genetically engineered mouse model of HCC (26). Approximately one-third of HCCs have Wnt/β-catenin mutations (27). A recent study found that advanced HCCs with Wnt/β-catenin mutations were resistant to immune checkpoint inhibitors and were associated with inferior prognosis (28). In this case, β-catenin expression was found in the lung-remaining metastatic tumor, which may cause resistance to sintilimab through the Wnt/β-catenin signaling pathway (**Supplementary Figure S1**). The therapeutic options for HCC are diverse, and comprehensive treatment is always required, especially for recurrent tumors. For the advanced HCCs, systemic therapies, including tyrosine kinase inhibitor-based targeted therapy and ICI-based immunotherapy, are first considered. However, it is always hard to achieve tumor CR by single or combinational systemic therapies. This case provided the new therapeutic strategy that after effective immunotherapy, there will be a new potential to perform radical surgery for the remaining lesions, which may obtain CR and finally increase the curable rate. However, the evidence level of a single case report is actually low, and high-quality, large-scale studies with long-term follow-up are truly needed.

This case demonstrated the existence of both spatial heterogeneity and temporal heterogeneity of liver cancer in the same patient, which results in the heterologous effects of anti-PD-1 treatment. This case also provides a promising option for multiple metastatic tumors that through a combination of immune therapy and surgery, there is still the potential to achieve an outcome of CR.

## Material and methods

Hematoxylin and eosin staining (H&E) and immunohistochemistry (IHC) were performed according to the common protocols in the Department of Pathology in our hospital. Staining was performed using the following antibodies: PD-L1 (Clone 22C3; Agilent Technologies, CA, USA), CD8 (Clone SP16; Novocastra, Newcastle upon Tyne, UK), CD3 (Clone EP41; ZSGB-BIO, Beijing, China), CD4 (Clone SP35; MXB, Fuzhou, China), CD57 (Clone NK-1 (RUO); BD Biosciences, NJ, USA), CD20 (Clone SP32; Abcam, Cambridge, UK); CD68 (Clone C68/684; Abcam, Cambridge, UK), CD11c (Clone Polyclonal; Affinity Biosciences, OH, USA); Foxp3 (Clone D2W8E; CST, MA, USA) and β-catenin (Clone Polyclonal; IL, USA).

## Data availability statement

The raw data supporting the conclusions of this article will be made available by the authors, without undue reservation.

## Ethics statement

The studies involving human participants were reviewed and approved by the center for ethics in the First Affiliated Hospital of Zhejiang University. Written informed consent was obtained from the patient for publication of this manuscript and for any accompanying clinical information and images. The patients/participants provided their written informed consent to participate in this study.

## Author contributions

All authors contributed substantially to the development of the study. TB Liang, YY Bao and L Wen designed the article. YY Bao and L Wen developed the idea and wrote the first draft of the manuscript. W Chen and JH Zhao contributed to figures and analysis. YX Yang, T Wei and J Zhang provided the study reagents and performed the supplementary experiments. All the authors read, modify and approved the final manuscript.

## Funding

The study was supported by the National Natural Science Foundation of China (NSFC 81902409) and the Natural Science Foundation of Zhejiang Province (Grant No. Y21H030014).

## Conflict of interest

The authors declare that the research was conducted in the absence of any commercial or financial relationships that could be construed as a potential conflict of interest.

## Publisher’s note

All claims expressed in this article are solely those of the authors and do not necessarily represent those of their affiliated organizations, or those of the publisher, the editors and the reviewers. Any product that may be evaluated in this article, or claim that may be made by its manufacturer, is not guaranteed or endorsed by the publisher.

## Supplementary material

Supplementary Figure 1 The tumor microenvironment of primary and recurrent tumors. In the primary tumor in the liver, IHC showed positive β-catenin expression and infiltration of T cells, B cells, macrophages, and natural killer cells. In the metastasis in the lung after Sintilimab treatment, IHC showed positive β-catenin expression and infiltration of T cells, B cells, macrophages, natural killer cells, and a few regulatory T cells. CD3, T cell marker; CD4, T helper cell marker; CD11c, dentritic cell marker; CD20, B cell marker; CD 57, natural killer cell marker; CD68, macrophage marker; Foxp3, regulatory T cell marker. Foxp3 staining is indicated by red arrows.

## Abbreviations

ICI, immune checkpoint inhibitor; PD-1, programmed cell death receptor-1; HCC, hepatocellular carcinoma; AFP, α-fetoprotein; TACE, trans-hepatic arterial chemotherapy and embolization; CT, computerized tomography.

## References

[1] Zhang Q Lou Y Yang J Wang J Feng J Zhao Y. Integrated multiomic analysis reveals comprehensive tumour heterogeneity and novel immunophenotypic classification in hepatocellular carcinomas. Gut (2019) 68:2019–31. doi: 10.1136/gutjnl-2019-318912 PMC6839802 31227589

[2] McGranahan N Swanton C. Clonal heterogeneity and tumor evolution: Past, present, and the future. Cell (2017) 168:613–28. doi: 10.1016/j.cell.2017.01.018 28187284

[3] Sharma P Hu-Lieskovan S Wargo JA Ribas A. Primary, adaptive, and acquired resistance to cancer immunotherapy. Cell (2017) 168:707–23. doi: 10.1016/j.cell.2017.01.017 PMC5391692 28187290

[4] Hoy SM. Sintilimab: First global approval. Drugs (2019) 79:341–6. doi: 10.1007/s40265-019-1066-z 30742278

[5] Ren Z Xu J Bai Y Xu A Cang S Du C. Sintilimab plus a bevacizumab biosimilar (IBI305) versus sorafenib in unresectable hepatocellular carcinoma (ORIENT-32): a randomised, open-label, phase 2–3 study. Lancet Oncol (2021) 22:977–90. doi: 10.1016/S1470-2045(21)00252-7 34143971

[6] Dagogo-Jack I Shaw AT. Tumour heterogeneity and resistance to cancer therapies. Nat Rev Clin Oncol (2018) 15:81–94. doi: 10.1038/nrclinonc.2017.166 29115304

[7] Ferronato M Lalanne C Quarneti C Cevolani M Ricci C Granito A. Paraneoplastic anti-Tif1-gamma autoantibody-positive dermatomyositis as clinical presentation of hepatocellular carcinoma recurrence. J Clin Trans Hepatol (2022) 000:000–0. doi: 10.14218/JCTH.2021.00573 PMC9647099 36406323

[8] Marusyk A Polyak K. Tumor heterogeneity: causes and consequences. Biochim Biophys Acta (2010) 1805:105–17. doi: 10.1016/j.bbcan.2009.11.002 PMC2814927 19931353

[9] Casadevall D Clave S Taus A Hardy-Werbin M Rocha P Lorenzo M. Heterogeneity of tumor and immune cell PD-L1 expression and lymphocyte counts in surgical NSCLC samples. Clin Lung Cancer (2017) 18:682–691 e685. doi: 10.1016/j.cllc.2017.04.014 28549836

[10] Duverger L Osio A Cribier B Mortier L De Masson A Basset-Seguin N. Heterogeneity of PD-L1 expression and CD8 tumor-infiltrating lymphocytes among subtypes of cutaneous adnexal carcinomas. Cancer Immunol Immunother (2019) 68:951–60. doi: 10.1007/s00262-019-02334-8 PMC11028315 30953116

[11] Garcia-Diez I Hernandez-Ruiz E Andrades E Gimeno J Ferrandiz-Pulido C Yebenes M. PD-L1 expression is increased in metastasizing squamous cell carcinomas and their metastases. Am J Dermatopathol (2018) 40:647–54. doi: 10.1097/DAD.0000000000001164 29742559

[12] Kim H Kwon HJ Park SY Park Y Park E Chung JH. Clinicopathological analysis and prognostic significance of programmed cell death-ligand 1 protein and mRNA expression in non-small cell lung cancer. PloS One (2018) 13:e0198634. doi: 10.1371/journal.pone.0198634 29856861 PMC5983554

[13] Balar AV Castellano D O’Donnell PH Grivas P Vuky J Powles T. First-line pembrolizumab in cisplatin-ineligible patients with locally advanced and unresectable or metastatic urothelial cancer (KEYNOTE-052): a multicentre, single-arm, phase 2 study. Lancet Oncol (2017) 18:1483–92. doi: 10.1016/S1470-2045(17)30616-2 28967485

[14] Reck M Rodriguez-Abreu D Robinson AG Hui R Csoszi T Fulop A. Pembrolizumab versus chemotherapy for PD-L1-Positive non-Small-Cell lung cancer. N Engl J Med (2016) 375:1823–33. doi: 10.1056/NEJMoa1606774 27718847

[15] Smith KN Llosa NJ Cottrell TR Siegel N Fan H Suri P. Persistent mutant oncogene specific T cells in two patients benefitting from anti-PD-1. J Immunother Cancer (2019) 7:40. doi: 10.1186/s40425-018-0492-x 30744692 PMC6371497

[16] Ribas A Hu-Lieskovan S. What does PD-L1 positive or negative mean? J Exp Med (2016) 213:2835–40. doi: 10.1084/jem.20161462 PMC5154949 27903604

[17] Sharma P Callahan MK Bono P Kim J Spiliopoulou P Calvo E. Nivolumab monotherapy in recurrent metastatic urothelial carcinoma (CheckMate 032): a multicentre, open-label, two-stage, multi-arm, phase 1/2 trial. Lancet Oncol (2016) 17:1590–8. doi: 10.1016/S1470-2045(16)30496-X PMC5648054 27733243

[18] Xing X Guo J Ding G Li B Dong B Feng Q. Analysis of PD1, PDL1, PDL2 expression and T cells infiltration in 1014 gastric cancer patients. Oncoimmunology (2018) 7:e1356144. doi: 10.1080/2162402X.2017.1356144 29399387 PMC5790386

[19] Yagi T Baba Y Ishimoto T Iwatsuki M Miyamoto Y Yoshida N. PD-L1 expression, tumor-infiltrating lymphocytes, and clinical outcome in patients with surgically resected esophageal cancer. Ann Surg (2019) 269:471–8. doi: 10.1097/SLA.0000000000002616 29206673

[20] Teng MW Ngiow SF Ribas A Smyth MJ. Classifying cancers based on T-cell infiltration and PD-L1. Cancer Res (2015) 75:2139–45. doi: 10.1158/0008-5472.CAN-15-0255 PMC4452411 25977340

[21] Granito A Muratori L Lalanne C Quarneti C Ferri S Guidi M. Hepatocellular carcinoma in viral and autoimmune liver diseases: Role of CD4+ CD25+ Foxp3+ regulatory T cells in the immune microenvironment. World J Gastroenterol (2021) 27:2994–3009. doi: 10.3748/wjg.v27.i22.2994 34168403 PMC8192285

[22] Yi M Jiao D Xu H Liu Q Zhao W Han X. Biomarkers for predicting efficacy of PD-1/PD-L1 inhibitors. Mol Cancer (2018) 17:129. doi: 10.1186/s12943-018-0864-3 30139382 PMC6107958

[23] Finn RS Ryoo BY Merle P Kudo M Bouattour M Lim HY. Pembrolizumab as second-line therapy in patients with advanced hepatocellular carcinoma in KEYNOTE-240: A randomized, double-blind, phase III trial. J Clin Oncol (2020) 38:193. doi: 10.1200/JCO.19.01307 31790344

[24] Kwee SA Tiirikainen M. Beta-catenin activation and immunotherapy resistance in hepatocellular carcinoma: mechanisms and biomarkers. Hepatoma Res (2021) 7:8. doi: 10.20517/2394-5079.2020.124 33553649 PMC7861492

[25] Spranger S Bao R Gajewski TF. Melanoma-intrinsic beta-catenin signalling prevents anti-tumour immunity. Nature (2015) 523:231–5. doi: 10.1038/nature14404 25970248

[26] Ruiz de Galarreta M Bresnahan E Molina-Sanchez P Lindblad KE Maier B Sia D. Beta-catenin activation promotes immune escape and resistance to anti-PD-1 therapy in hepatocellular carcinoma. Cancer Discovery (2019) 9:1124–41. doi: 10.1158/2159-8290.CD-19-0074 PMC6677618 31186238

[27] Zucman-Rossi J Villanueva A Nault JC Llovet JM. Genetic landscape and biomarkers of hepatocellular carcinoma. Gastroenterology (2015) 149:1226–1239 e1224. doi: 10.1053/j.gastro.2015.05.061 26099527

[28] Harding JJ Nandakumar S Armenia J Khalil DN Albano M Ly M. Prospective genotyping of hepatocellular carcinoma: Clinical implications of next-generation sequencing for matching patients to targeted and immune therapies. Clin Cancer Res (2019) 25:2116–26. doi: 10.1158/1078-0432.CCR-18-2293 PMC6689131 30373752

