# Supplementary figures and images for: Case report: A case of heterogeneity of the antitumor response to immune checkpoint inhibitors in a patient with relapsed hepatocellular carcinoma
Source: Front Oncol. 2022 Jul 29;12:899811. doi: 10.3389/fonc.2022.899811 (PMC9372450; doi:10.3389/fonc.2022.899811)

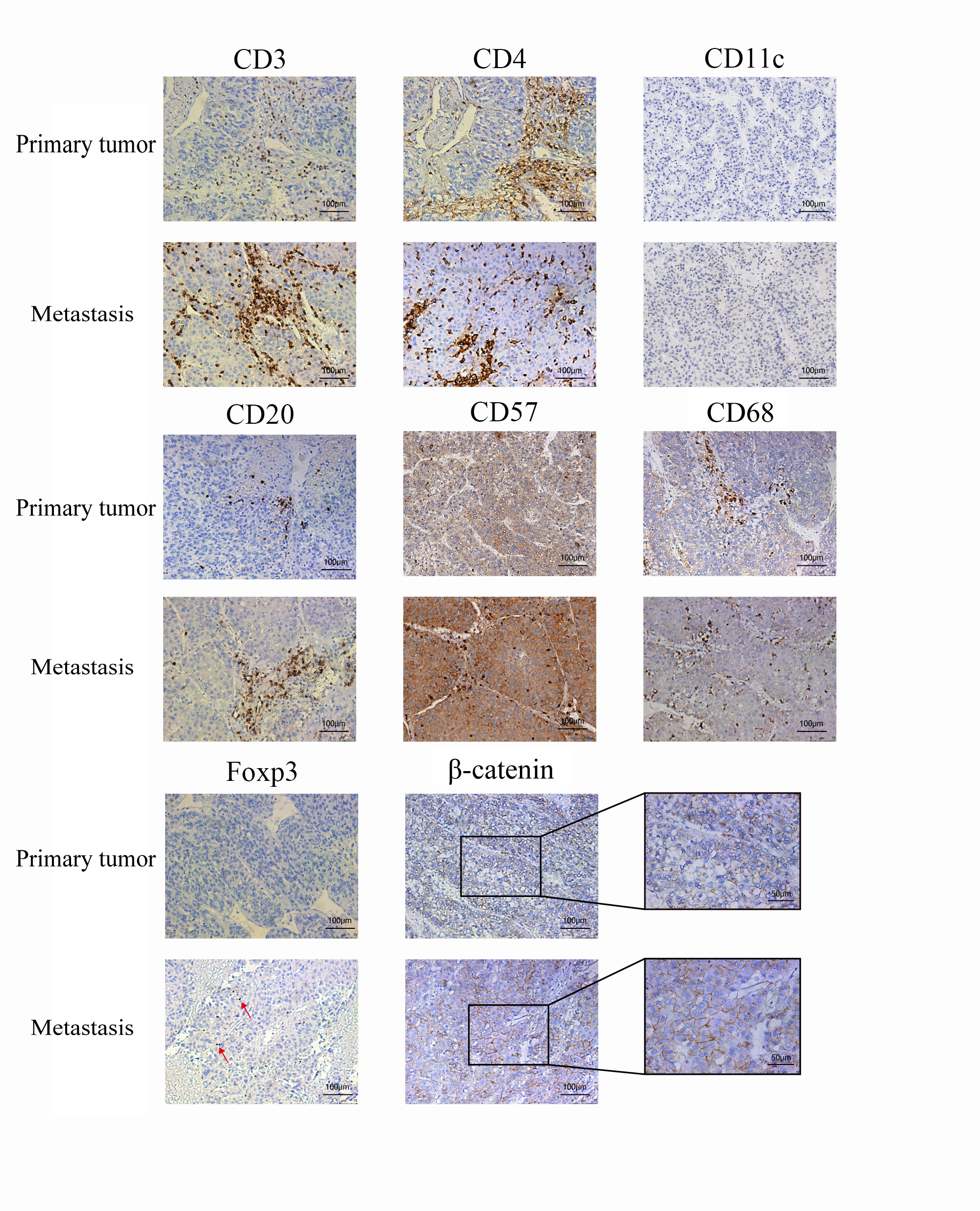

Supplement: Supplementary Figure 1 — The tumor microenvironment of primary and recurrent tumors. In the primary tumor in the liver, IHC showed positive β-catenin expression and infiltration of T cells, B cells, macrophages, and natural killer cells. In the metastasis in the lung after Sintilimab treatment, IHC showed positive β-catenin expression and infiltration of T cells, B cells, macrophages, natural killer cells, and a few regulatory T cells. CD3, T cell marker; CD4, T helper cell marker; CD11c, dentritic cell marker; CD20, B cell marker; CD 57, natural killer cell marker; CD68, macrophage marker; Foxp3, regulatory T cell marker. Foxp3 staining is indicated by red arrows. [file Image_1.tif]
